# “SALOME gave my dignity back”: The role of randomized heroin trials in transforming lives in the Downtown Eastside of Vancouver, Canada

**DOI:** 10.3402/qhw.v9.23698

**Published:** 2014-03-13

**Authors:** Ehsan Jozaghi

**Affiliations:** School of Criminology, Simon Fraser University, Burnaby, Canada

**Keywords:** SALOME, prescribed heroin, Downtown Eastside, injection drug users, overdose

## Abstract

Although numerous studies on heroin-assisted treatment (HAT) have been published in leading international journals, little attention has been given to HAT’s clients, their stories, and what constitutes the most influential factor in the treatment process. The present study investigates the role of HAT in transforming the lives of injection drug users (IDUs) in Vancouver, Canada. This study is qualitative focusing on 16 in-depth interviews with patients from the randomized trials of HAT. Interviews were transcribed verbatim and analyzed thematically using NVivo 10 software. The findings revealed a positive change in many respects: the randomized trials reduce criminal activity, sex work, and illicit drug use. In addition, the trials improved the health and social functioning of its clients, with some participants acquiring work or volunteer positions. Many of the participants have been able to reconnect with their family members, which was not possible before the program. Furthermore, the relationship between the staff and patients at the project appears to have transformed the behavior of participants. Attending HAT in Vancouver has been particularly effective in creating a unique microenvironment where IDUs who have attended HAT have been able to form a collective identity advocating for their rights. The result of this research points to the need for continuation of the project beyond the current study, leading toward a permanent program.

The Downtown Eastside (DTES) of Vancouver, Canada, was the center of North America’s open illicit drug scene for decades (Jozaghi, 2012, 2014; Kerr, MacPherson, & Wood, 2008). During the mid-1990s, the area’s problems escalated when several factors, such as an increase in the availability of powder cocaine, lack of social housing, and deinstitutionalization resulted in a multiple epidemic of injection drug use, HIV, and drug overdose deaths (Jozaghi, 2012). What differentiated Vancouver from other North American cities was the magnitude of the outbreak; for example, between 1990 and 2000 HIV prevalence rate among injection drug users (IDUs) rose from 4% to up to 40% (O’Shaughnessy, Hogg, Strathdee, & Montaner, 2012). At the same time, a growing overdose epidemic was taking place within the IDU population in Vancouver, with 1200 deaths occurring between 1992 and 2000 (Kerr et al., 2008).

According to Kerr, Small, Hyshka, Maher, and Shannon (2013), Vancouver “has long been home to dual epidemics of heroin injection and heroin related overdose” (p. 108). In fact, there have been numerous public warnings regarding heroin-related deaths in Vancouver (Kerr et al., 2013). In addition to overdose deaths, there are numerous costs to the health care, criminal justice, and welfare systems when dealing with the numerous challenges that opioid and heroin users pose for the government (Gartry, Oviedo-Joekes, Laliberte, & Schechter, 2009). A recent Auditor General’s report estimated illicit drug use to cost Canadian tax payers an estimated $5 billion annually (Miller et al., 2004). According to the Auditor General’s report, the societal cost of untreated heroin addiction exceeds $45,000 per person per year (Status Report, 2008). Since the 1960s in Canada, methadone maintenance therapy (MMT) has been the main substitute for heroin and opioid addiction (Dole & Nyswander, 1965).

MMT has been shown to be effective in decreasing the incidence of illicit opioid use and cravings; additionally, in some cases, MMT may lead to abstinence (Gartry et al., 2009). However, several factors have suggested the inefficiency of MMT for some users. For example, “inadequate methadone doses, user fees, punitive urine testing, and a lack of high associated with MMT” have all been cited (Gartry et al., 2009, p. 2). Additionally, MMT is not effective in all cases; between 15 and 25% of patients do not respond to MMT, especially among long-term, high-risk patients (van den Brink et al., 2003; Mino, Page, Dumont, & Broers, 1998).

In response to the growing number of addicts who do not respond to traditional MMT programs, numerous European countries have started experimenting with pharmaceutical heroin. Since 1926, addiction treatment in the United Kingdom has included heroin prescription (Gartry et al., 2009). In the Netherlands and Switzerland, heroin-assisted treatment (HAT) clinics have been in operation since the 1990s, and Germany is in the process of creating a centralized system for diamorphine (Gartry et al., 2009). The scientific evaluation of HAT has been very positive in European countries (Gartry et al., 2009).

Canada has also taken notice of the success of the HAT program in Europe. In September 1998, the first North American Opiate Medication Initiative (NAOMI) working group was formed (Gartry et al., 2009). Recruitment in Montreal and Vancouver began in March 2005 and ended in April 2007 (Gartry et al., 2009). The 12-month program ended in June 2008, with 251 of the most marginalized and chronic opiate users entering the program (Gartry et al., 2009). Evaluation of the NAOMI, according to numerous peer-reviewed studies, has been positive (Bohdan et al., 2012; Nosyk et al., 2010; Kahan, Srivastava, & Conway, 2011).

In the interim, another set of trials, the Study to Assess Long-term Opioid Medication Effectiveness (SALOME), has begun to test whether people affected by chronic opioid addiction, who are not improving sufficiently from other treatments, will benefit from hydromorphone (a licensed medication), and whether hydromorphone is as effective in treating addiction as diacetylmorphine (Mickleburgh, 2011; Oviedo-Joekes et al., 2009). SALOME began active recruitment in December 2011 (Boyd, 2012). After 1 year of participating in the program, participants were encouraged to take part in conventional treatment, such as MMT and detox, during a period of transition (Boyd, 2012). The current study seeks to determine the transformative role of HAT in the lives of IDUs. Specifically, this study will account for (1) experience prior to the SALOME trial; (2) injection behavior of clients before enrolling in the programs; (3) injection behavior post-enrollment into SALOME; (4) health status before and after the enrollment; (5) activism in the community; and (6) an open discussion regarding how the program was successful. This is the first qualitative study of SALOME. It is believed that participants’ accounts generated by qualitative narratives create opportunities for discovering a rich detailed description. This study will show the behavior of participants in the SALOME project in the DTES to be shaped by a complex interplay of individual and social factors, which are not amenable to quantitative measurements (Rhodes & Treloar, 2008).

## Methods

### Participants

This study was approved by Simon Fraser University’s Research Ethics Board (study No.: 2013s0279). Beginning in May 2013, participants living in the DTES, and those who in the previous week had attended SALOME, were recruited to participate in the study. Participants were eligible for the study if they were 19 years or older and provided oral consent. To protect confidentiality, all identifying information was removed from interview transcripts and all names used in this paper are pseudonyms.

The study was facilitated through recruitment of participants through purposive sampling via key informants (Jozaghi & Andresen, 2013; Jozaghi, 2013). The researcher and the key informants had a pre-existing relationship that was established during the researcher’s previous volunteer work in the DTES.^1^ The first key informant was one of the co-founders of NAOMI Patients Association (NPA). The researcher had met her through the Vancouver Area Network of Drug Users (VANDU). The second key informant was a volunteer at Washington’s Needle Depot in the DTES, where the researcher also volunteered for 2 years. To protect the confidentiality of participants, this research only reported the demographic characteristics of the participants in a quantitative manner (Table I).

**Table I:** Characteristics of the sample of SALOME in Vancouver.

	*n*	%
Age		
20–30	1	6.25
31–40	1	6.25
41–50	9	56.25
51–61	5	31.25
Gender		
Male	9	56.25
Female	7	43.75
Ethnicity		
Caucasian	12	75.0
First Nation	4	25.0
Sexual orientation		
Heterosexual	15	93.75
Homosexual	1	6.25
Number of injection per day (*X*)		
*X*=0 (oral)	5	31.25
2 <*x*≤3	9	56.25
*x*>3	2	12.5
Years of injection (*Y*)		
*Y*≤10	2	12.5
10<*Y*≤20	4	25.0
*Y*>20	10	62.5
Medical condition		
Nil	3	18.75
Hepatitis B	1	6.25
HCV	10	62.5
Mental illness	1	6.25
Mental illness and HCV	1	6.25
Dosage per day in mL (*µ*)		
10<*µ*≤20	1	6.25
20<*µ*≤200	2	12.5
20<*µ*≤500	5	31.25
500<*µ*≤1000	8	50.0
High school education		
Yes	6	37.5
No	10	62.5
Sex trade in the last 6 months		
Yes	1	6.25
No	15	93.75
Money/drugs for sex in the last 6 months		
Yes	3	18.75
No	13	81.25
Relationship status		
Single/divorced	14	87.5
Married/common law	2	12.5
Months in the program (*α*)		
0<*α*≤2	2	12.5
2<*α*≤6	3	18.75
*α*>6	11	68.75
Principal dwelling place		
Private residence or other’s house	15	93.75
Street/shelter	1	6.25
Criminal record		
Yes	15	100.0
No	0	0.0
Unemployed		
Yes	15	93.75
No	1	6.25
Years of residing in the DTES (*Z*)		
2≤*Z*≤15	11	68.75
15<*Z*≤25	4	25.0
*Z*>25	1	6.25

Participants were told by key informants that the interviewer was a doctoral student. None of those who were invited to participate refused. In fact, the referral by the key informants allowed the participants to feel comfortable and open up more easily about their experiences. Sixteen purposefully chosen IDUs were found to be eligible for this study. When the interview took place, six participants had been in the program for more than 6 months, three participants had been in the program for 4 months, and the remainder of the participants had been in the program for 2 months.

### Procedure

Open-ended, semi-structured interviews were conducted through the use of an interview guide. Using Dragon Naturally Speaking software, all the transcripts were transcribed verbatim. Apart from a few key questions, participants were encouraged to discuss the issues as they saw fit. Each interview took on a story of its own. All participants indicated genuine interest in the interview by using individual examples and had unique stories to shape the direction of the interview. As suggested by Berg (2009), deviations were explored in all the interviews, because topics arose from natural conversation.

A digital voice recorder (Olympus WS-700M) and a small notebook were used to allow the researcher to jot down key words or phrases during the interview. This strategy allowed the researcher to pay attention to the participants and take occasional notes to retain the flow of the interview. Also, the researcher took time to record field notes after each interview, including impressions of the interview, questions to ask in future interviews, and interesting themes to consider during the analysis. This strategy allowed for the recording of rich details about each interview.

### Data analysis

After reviewing the qualitative data (notes and transcripts), NVivo 10 software was used for thematic analysis. Before importing the data in NVivo 10, interviews were listened to several times.

By relying on NVivo 10, themes were coded as nodes. The first stage involved in this process was open coding. During this stage, through deductive and general observation using a word frequency query, “free” nodes were created which resulted in more specific categories. Free nodes were created after key words—identified during the world frequency query—were searched in the transcript. The researcher grouped information into smaller sections at this stage, and then provided a descriptive name for each.

For example, 19 free nodes were identified and labeled as the following: (1) relationships; (2) negative effects; (3) beginning; (4) oral; (5) health; (6) methadone; (7) NAOMI/SALOME history; (8) changes in behavior; (9) dosage; (10) overdose; (11) services; (12) transition; (13) normal life; (14) life prior to HAT; (15) change; (16) activism; (17) crime; and (18) frustration.

Through an inductive search, a “tree” node was created for general themes, in which the sections were grouped into common categories. This process was performed iteratively until all interview transcripts had been accounted for, and all new or developing themes had reached a saturation point. To avoid overrepresentation of themes, the most dominant themes were identified through the organization and analysis of the data.

In addition, the coding was conducted via color highlighters. Analyzing the data via highlighters was a rather natural process and resembled “the spiralling research approach” (Berg, 2009, p. 26). As I was transcribing the interviews and later analyzing them via highlighters, it did not take long to realize possible themes that were emerging and what these themes may mean in terms of the existing literature. Further, during the writing process I was still engaged in the process of coding, and analysis, as I was thinking of new themes, reframing themes, and revisiting the tapes and transcripts for elaboration or conformation.

## Findings

To illustrate the central themes that emerged in the cross-case analysis, excerpts from the qualitative interviews are presented below. Dominant themes included the following: activism, health, social tenure, and crime. Although the systematic analysis with NVivo 10 software confirmed a broad overlap across thematic areas among study participants, data were analyzed from each participant independently.

### Activism

The SALOME project could not be comprehended without considering the NAOMI project’s influence on many participants after the program ended. Although participants praise the NAOMI project for their efforts to provide heroin prescription in the DTES, they also remain critical of the program for its lack of exit strategy. For example, according to Lila:

Once NAOMI study ended I was devastated …. The ethics behind what they did is wrong … out of the 97 of us that were on the needle side 13 people died … that’s like 15% of people died because they couldn’t cope with a life outside anymore … people were just devastated they could not cope with the hustling again with selling their bodies or committing crime. After NAOMI ended a lot of girls turned into the street to support their habit. And so many guys started the hustling again and turned into the life of crime.

According to Boyd and NAOMI Patients Association (2013), the NAOMI project’s conclusion was questionable on many issues, including its failure to provide a permanent program, the absence of an exit strategy, and lack of informed consent. Boyd and NAOMI Patients Association (2013) also point to the Helsinki Declaration on international ethical standards where “at the conclusion of a study, every patient … should be assured access to the best … methods identified by the study” (p. 7). According to Lila, the failure of the NAOMI project to ensure an exit strategy and the approval of a similar study to be conducted in the DTES ignited the creation of an advocacy group called the NPA:

The people who were on NAOMI decided to form a group and we started talking and we heard SALOME was coming in place and we were not going to stop and let them do the same thing that they did to us. This time it’s going to be 360 people who were going to be on this program. And that’s why we found the NPA. We decided to recruit people who were known to us and bring them to the meetings at VANDU. They wanted to make sure that SALOME gets the proper procedure and so that they’re not going to screw us all over again. We learned over the years that peer groups work.

Currently, members of NPA have weekly meetings at the VANDU, where they discuss the issues facing the participants at SALOME (Boyd & NAOMI Patients Association, 2013). To achieve their goals, members of NPA have been politically active in their community. As Lila describes:

And as part of [NPA] we went to rallies … we went to Providence Health, we went to the Vancouver Coastal Health meetings so they could hear our voice: not just academics, but also people like us, junkies on the street that are affected by these programs every day. And it worked, they listened to us. At first it was only 4 to 5 of us. But within six months we were able to get hold of 40 people who were part of the project. The group got bigger by that time. … We have gone through different conferences talking about our cause.

Some members of NPA are not only dedicated to and focused on supporting their members, but they are also involved in advocacy work and political empowerment in the DTES. The NPA, according to Boyd and NAOMI Patients Association (2013), is also involved in education of its members and the general public. Therefore, after advocating for their rights since January 2011, NPA has recently been successful in forcing the government to extend the program for those who will remain on the oral use of hydromorphone.

### Increased health

All 16 participants indicated that their health and well-being has improved drastically since enrolling in SALOME. Many of the participants have been able to improve their nutrition, and to reduce their stress and risky behavior, such as sex work. For some of the participants such as Monique, SALOME has even allowed her to look for a job:

Life is less stressful, I don’t have to worry about my next fix I don’t have to worry about getting sick. It has enabled me to do other things like look for a job or volunteer work in the Downtown Eastside. Before I had to go, get ready to go to work every night on the Street and I don’t have to do that anymore.

Miller et al.’s (2004) study also demonstrated that a medical heroin prescription program has the potential to moderately increase employment rates for these DTES residents. For some of the participants such as Joe, the program has helped them to recover from long term illness and the struggle with addiction:

My health was really bad … I was always in pain and constantly in and out of hospital. And when you’re down and shit like that and you’re sick and in pain all the time, it’s a shitty way to live your life. This was the God work for me to get on the program. Don’t necessarily get high on the program but get well, to manage my pain now way better than before. Now I have a lot more energy before I was sick all the time. All of a sudden I have that energy, I have the drive, I have that motivation.

Previous studies have also demonstrated that heroin prescription programs are able to reduce hospital stay times and the number of emergency room visits (Miller et al., 2004; van den Brink et al., 2003). The study by Nosyk et al. (2010) also showed that the motivational status of patients was instrumental in creating a more favorable response to treatment. Many of the participants indicated that because of SALOME, they have been able to change their life around and reduce daily stress. This is particularly important for participants such as Nicole who suffered from a mental illness:

SALOME gave my dignity back. Didn’t make me feel like … I was a loser …. Now I have a better relationship with my adult children. All of a sudden when you realize you’re an addict and you hide it, and you finally get the chance to tell somebody. And this is what SALOME has done for me. And they allowed me and encouraged me to tell my story. It’s hard to get used to it at first that your opinion counts. Give me a chance to do something about my life and finally change my life for the better. I do think if it wasn’t for SALOME at this point in my life I would be very depressed. I used to suffer from depression.

According to Small and Drucker (2006), prescription heroin programs reach a refractory group of addicts who, through their interaction with staff and doctors in the clinics, are able to improve their health status. Furthermore, as stated above, heroin prescription trials enable and empower their clients to contact their families (Small & Drucker, 2006).

### Social tenure

For all participants, the relationships they have been able to establish with the social workers, nurses, and doctors at SALOME have enabled them to move beyond their addiction needs. One participant, Arthur, received much needed help for his hepatitis C (HCV) condition:

They helped me to get full dietary for my Hep C. With the help of SALOME I had a lot of dental work done …. And they helped me … to get my life get on track. SALOME helps me in regards to my use, and addiction. I was fixing about eight points a day^2^ where today I only fix one point a day. I feel like that I accomplished so much since I’ve been on this program. They are decreasing my dose right now where I’m hoping by the end of it, I will be able to go on detox and quit using drugs.

Previous research (Miller et al., 2004) supports this study’s findings that heroin prescription programs lead to reduced drug use. Furthermore, European studies, including ones from Switzerland, Germany, Spain, and the Netherlands, have shown that opiate addicts prescribed heroin under supervision have successfully reduced their drug use (Gartry et al., 2009). Many of the participants indicate that their life has been transformed for the better due to the kind relationships the staff has fostered with their clients. For instance, according to Ashley:

SALOME people help us to get housing, we got evicted from our place where we lived and they went to arbitration with us. They did our taxes. They even came to the Ministry so I could see my son. And if I go on two different appointments they stay with me and calm me down because I have an anxiety disorder and talked with me because of my anxiety problem …. And I come from a very disadvantaged family, where there was a lot of incest in my family, and the person that I felt comfortable talking about my experience was at SALOME with the counselors and the doctor there and they were very supportive of me.

Oviedo-Joekes et al.’s (2008) study also suggests that the NAOMI cohort comprised a higher proportion of marginalized populations, such as those residing in unstable housing. The relationship building efforts of the staff could be one of the influential factors contributing to the improved social functioning of participants. Additionally, nurses at the facility have been successful in reducing the possibility of drug overdose death with participants at the facility. For example, according to Monica:

I was Narcaned for the first time at SALOME, and if I wasn’t at SALOME, I’d be dead because I usually fix alone …. Also my ex-husband overdosed numerous times and they saved his life too …. They also gave me training to use Narcan. The pharmacist at SALOME actually gave us a speech and told us how to use it. People drop in the Downtown Eastside, people pretend that they’re helping them, but they are robbing them instead.

Injecting outside of SALOME carries association of fear, overdose, and death, which reinforce the security and safety that participants have come to associate with the facility. In fact, all the participants who have used SALOME have seen an overdose or have experienced an overdose at SALOME, and all of them agree that the quick responses of nurses to an overdose situation has reduced the possibility of overdose deaths. Furthermore, the relationship between patients, nurses, social workers, and doctors at the facility has facilitated harm reduction education.

### Diminished criminality

The most common theme across the analysis was related to illicit behavior of participants prior to enrolling into SALOME program. As shown in Table I, all the participants had some sort of reported criminal record. For female participants, such activities usually involved sex work and drug dealing. For male participants, they usually involved breaking-and-entering, shoplifting, drug dealing, and robbery. According to Michael, criminal activity prior to SALOME is tied to the vicious cycle of addiction or as he puts it, “the dope sickness”^3^:

My main concern every day, all day was making sure I was going to be able to get some kind of opiates right …. So I was constantly looking to make the money for my dope, and it just consumes you. Doesn’t matter about anything else, the only biggest concern is to get the money so you wouldn’t get dope sick. My life was a fucking mess because the only thing I cared about was dope sickness. It was all about getting that money. It wouldn’t matter, I would stay awake for days so I would be able to get the money. As soon as you pass that point that you’re sick, then you’re scored …. Sometimes it’s friends; other times, desperation kicks in. And when you’re desperate, anything goes.

The Vancouver Board of Trade estimated in 2005 that property crime was costing the city’s taxpayers $125 million annually (Park, 2009). The majority of property crimes, according to the Vancouver Police Department, are committed by addicts who can require $100 or more daily for purchasing drugs (O’Conner, 2009). The DTES of Vancouver is the crime’s “ground zero” where in 2008 the area accounted for 16% of sexual assaults, 22% of robberies, and 34.5% of serious assaults (O’Conner, 2009). However, SALOME altered the criminogenic behavior of participants such as Rob:

I used to do a lot of debt collection for a lot of dealers down here—that was my job before SALOME. But now my job is freelance reporter in the Downtown Eastside community. I was a very violent person but now I have taken a different route and I have been able to manage my anger and work through my problems. So SALOME has allowed me to take a step back and say to myself: “There is something that needs to be done.” And that’s how I’ve been able to change my life, because SALOME has provided the stability and security where I don’t have to hustle, hurt people and break into the cars to support my habit.

According to DeBeck et al.’s (2007) research, the costs associated with illicit drugs are “compelling IDUs, specifically those possessing markers of higher intensity addiction, to engage in prohibited income generating activities” (p. 50). However, as suggested by Rob above, once participants are enrolled in the SALOME program, they no longer feel the urge to commit criminal activity to support their habit. Some of the participants, such as Jack, have been able to not only reduce their illicit behavior, but turn their lives around:

I am not as aggressive as before because of SALOME. My personality is much more bearing. I don’t get hyper. I don’t have to get up early to go hustle. I am more calm and reserved. And because I don’t have to be rushed to make that extra buck, it gives you hope that people are not treating you as a disease and a piece of shit … I didn’t even like myself before the SALOME. But now I can look into a mirror and see a totally changed person. I like myself now. Talking to doctors and social workers at SALOME had affected me the most because they are not only giving you the drug, they also talk to you, they treat you like a human being …. They help you with better housing … [and] how to get healthy, and they are there for you if you just want to talk to someone.

Miller et al. (2004) estimate that medically prescribed heroin programs have the potential to decrease criminal activity, hospital, and emergency costs by $9650, a 63% reduction. According to Dr. Schechter, although the SALOME program may cost $7500 per addict, this is a bargain compared to the estimated $50,000 each untreated addict costs the health care system (Skelton, 2008).

## Discussion and conclusions

This paper set out to investigate whether SALOME was successful in improving the lives of its participants. Specifically, this study was interested in accounting for (1) experience prior to the SALOME trial; (2) injection behavior of clients before enrolling in the programs; (3) injection behavior post-enrollment into SALOME; (4) health status before and after the enrollment; (5) activism in the community; and (6) an open discussion regarding how the program was successful. The findings revealed a reported positive change in many respects: SALOME reduces criminal activity, sex work, and illicit drug use. In addition, SALOME has improved the health and social functioning of its clients, with some participants acquiring work or volunteer positions. Many of the participants have reconnected with their family members, an unlikely feat before SALOME.

Furthermore, the relationship between the staff and patients at SALOME has helped to transform the behavior of participants. The staff has been able to establish trust amongst the participants that has ultimately helped participants get much needed medical help for their undiagnosed medical conditions such as mental illness or endocarditis. The close bond and the relationship that exists between the staff and patients at SALOME has increased nursing, counseling care, and harm reduction education. The social workers at SALOME have also been able to improve the housing and financial situations of participants through social housing and income assistance. The findings of this study also suggest that in order for the benefits to continue, the program needs to become permanent, in order to avoid the same consequences as the NAOMI project.

The most prominent finding of this study has been the identification of social activism by IDUs who have attended the randomized trials. According to Capitanio and Herek (1999), drug users have received particularly harsh condemnation over the past century through labeling; IDUs, and drug users in general, are viewed as “dregs of society who steal to support their habit and pollute mainstream society with their chaotic behavior and drug related illness” (Hippel & Brener, 2012, p. 1030). Further, Harris and Fiske (2006) claim that IDUs are often dehumanized by others in society. These repressive measures and labeling of drug users can be understood through social constructionism. From a social constructionist perspective, meaning is assigned to an act or a behavior through a process of labeling by groups who seek to elicit a particular response. That is, something is defined as a social problem not based on the inherent nature of the behavior, but based on social responses to the behavior often instigated by the claim making of a particular group.

According to Derlega and Barbee (1999), once IDUs and drug users have been judged as socially illegitimate, they are excluded or ignored altogether. However, members of SALOME trials have been able to move beyond the demonizing rhetoric they endured as a consequence of the war on drugs. They have not only been able to change the dynamic of power relation that manifests in disproportionate suffering, but they have been able to reduce the misery that many IDUs experienced after the NAOMI project ended. Now they have a political voice through the NPA where they are able to discuss their weekly encounters with nurses, doctors, and social workers and raise their collective voice if they see any unfairness.

During their weekly meetings they are able to educate themselves and others about the benefits of HATs. They have been able to raise awareness about their cause through public demonstrations, writing poems, and inviting media to their meetings. Despite the challenges in the future, their collective identity will help them to push the boundaries and continuously strive to represent the concerns of SALOME patients. For example, with the help of the NPA and the Pivot Legal Society, some of the IDUs have filed a lawsuit against the Canadian federal government’s decision to stop doctors prescribing heroin to patients who have transitioned into oral diacetylmorphine (Woo, 2013).

Despite the noted findings above, the current study has several limitations that should be acknowledged. First, although this study reports many commentaries related to experiences, perspectives, and values of participants at SALOME, because of access and the time spent in the field, triangulation was limited. The researcher would have preferred to supplement many of the interviews with more observations, especially regarding interactions between the participants and staff within the SALOME location.

Second, although purposive sampling has shown to be instrumental, additional participants would ultimately be required. Consequently, all the participants referred to the researcher had similar viewpoints regarding the role of SALOME. Finally, despite this study’s attempts to reduce the social desirability effect—by reminding the participants that there are no right or wrong answers and avoiding leading questions—its influence on participants was unavoidable. Consequently, some positive responses in regard to SALOME can be attributed to the social desirability effect. Moreover, the way the interviewer asked the questions, directed the conversation, closed the conversation, determined what constituted a correct or complete answer, and paid the participants, may have influenced participants’ responses (Jozaghi, 2012). In addition, many of the participants were recruited from a clinical trial where considerable resources are put in and the “enthusiasm” factor may well have influenced the positive treatment outcome. Some studies have gone as far as suggesting that up to 14% of the difference between responses can be attributed to enthusiasm and the social desirability effect (Heerwing & McCabe, 2009).

In summary, the current study was meant to generate discussion around the controversial SALOME experiment in Vancouver which is still ongoing. The current study demonstrated some societal, health, and criminal justice benefits that have derived from the SALOME experiment. Furthermore, the present study provided a snapshot of benefits for those participants who not only have engaged in long term illicit drug use, but also have not benefited from effective methadone therapy. However, the most significant finding of this study lies in the activism of IDUs, who have organized themselves to advocate for their rights. Despite the challenges in the future, their collective identity will help them to push the boundaries and continuously strive to represent the concerns of SALOME patients. It is our hope that continuing qualitative research will provide some of the critical information needed for government and health policy makers to make informed decisions, thereby reducing the harms and costs of opiate addiction in our society.

## Conflict of interest and funding

The author has not received any funding or benefits from industry or elsewhere to conduct this study.

## Footnotes

1 The interviewer volunteered in the Washington’s Needle Depot in the DTES for 2 years.

2 This refers to 0.8 g of heroin, or eight doses of 0.1 g.

3 “Dope sickness” refers to opiate withdrawal symptoms.
